# Toxicity Effects and Mechanism of Chemical Stress on *Pomacea canaliculata*

**DOI:** 10.3390/biology15070529

**Published:** 2026-03-26

**Authors:** Huayang Zhou, Meiling Zou, Zhixiong Zhou, Chuanren Li

**Affiliations:** 1 MARA Key Laboratory of Sustainable Crop Production in the Middle Reaches of the Yangtze River (Co-Construction by Ministry and Province), College of Agriculture, Yangtze University, Jingzhou 434025, China; 18572393185@163.com (H.Z.); zml101063@163.com (M.Z.); 2 Hubei Key Laboratory of Waterlogging Disaster and Agricultural Use of Wetland, College of Agriculture, Yangtze University, Jingzhou 434025, China

**Keywords:** *Pomacea canaliculata*, niclosamide sulfate, fentin acetate, metaldehyde, enzyme activity, metabolomics

## Abstract

*Pomacea canaliculata*, as a significant invasive alien species, poses severe threats to agricultural development. Currently, chemical applications demonstrate notable efficacy in controlling this pest. However, metaldehyde exhibits overly singular toxicity towards *P. canaliculata*; niclosamide sulfate is not a molluscicide; and fentin acetate is a fungicide. Currently, these findings fail to elucidate the physiological and biochemical effects of the compounds after they enter the *P. canaliculata*’s body. In this study, we evaluated the toxicity of metaldehyde (ME), niclosamide sulfate (NS), and fentin acetate (FA) against *P. canaliculata* and analyzed the morphological and physiological changes in response to chemical stress. The results indicated that three chemicals exhibited potent molluscicidal activity, especially in the NS treatment group. After 12 h exposure to LC_50_ concentrations (48 h LC_50_), the surface area of livers was reduced significantly by 12.1%, 13.9%, and 2.8% compared to the control group, while the kidneys expanded significantly by 6.4%, 3.2%, and 16.7%, respectively. The heart showed marked enlargement by 152.1% and 44.2% under niclosamide sulfate and metaldehyde treatments. The pulmonary sac significantly contracted by 23.6% under niclosamide sulfate stress but expanded by 6.1% under fentin acetate exposure. The stomach enlarged significantly after niclosamide sulfate treatment, whereas it shrank by 2.1% and 5.7% under metaldehyde and fentin acetate treatments, respectively. Metabolomic analysis of liver tissues revealed 553, 99, and 585 differential metabolites compared to the control group, respectively. KEGG pathway enrichment analysis showed that the metabolism pathway, lysine degradation, and bile secretion are likely related to the response to chemical stress in *P. canaliculata*. Further examination showed a significant decrease in total protein content and the activities of malondialdehyde (MDA), acetylcholinesterase (AChE), superoxide dismutase (SOD), and catalase (CAT) under chemical stress. These findings enhance our understanding of the targeted mechanisms of molluscicides against *P. canaliculata.* Metaldehyde may exert neurotoxic effects on the *P. canaliculata*, while niclosamide sulfate may interfere with its respiratory system. Additionally, both chemicals affect metabolic pathways in the snail’s liver, including lipid metabolism and metabolic pathways associated with energy metabolism. These findings provide valuable insights for designing a novel snail control agent and formulating scientific management strategy.

## Simple Summary

The apple snail, *P. canaliculate*, is a highly destructive invasive species. Although chemical control measures have shown notable effectiveness, the underlying targeted mechanisms remain poorly understood. In this study, three chemical agents, including metaldehyde, niclosamide sulfate and fentin acetate, were applied for evaluating their molluscicidal efficacy and toxicity mechanism on *P. canaliculata*. The three agents exhibited strong molluscicidal activity and induced morphological changes in organs of *P. canaliculata*. Metabolomic analysis of liver identified 553, 99, and 585 differentially abundant metabolites following exposure to metaldehyde, niclosamide sulfate, and fentin acetate, respectively. Additionally, a decrease in total protein concentration was observed, along with significantly reduced levels of malondialdehyde and decreased activities of acetylcholinesterase, superoxide dismutase, and catalase. These findings underscore the targeted action and metabolic impact of the three agents on *P. canaliculata*, providing a foundation for the development of novel molluscicides and informing scientific strategies for the management of this invasive species.

## 1. Introduction

*Pomacea canaliculata*, commonly known as the apple snail or golden apple snail, is a large freshwater gastropod belonging to the family Ampullariidae. It is native to the Amazon River basin in South America [1]. In the 1980s, this species was introduced to the Taiwan region of China for aquaculture purposes. However, due to inadequate management, it rapidly proliferated across freshwater river systems throughout mainland China and was listed among China’s first 16 most hazardous invasive alien species in 2003 [2]. As a notorious agricultural pest, *P. canaliculata* poses a significant threat to grain production by voraciously feeding on rice seedlings and young plants [3]. Furthermore, its pronounced environmental adaptability, resistance to certain pesticides, and high reproductive output confer a competitive advantage in invaded ecosystems, enabling it to outcompete native species for resources such as water and habitat, thereby undermining biodiversity. Moreover, *P. canaliculata* also presents considerable public health risks. It serves as an intermediate host for several zoonotic parasites [4], including Echinostoma revolutum and Angiostrongylus cantonensis [5]. Research indicates that improper preparation, such as consuming *P. canaliculata* raw or undercooked, can lead to parasitic infections in humans through the ingestion of infective larvae [6].

Chemical control is currently the most efficient method for managing *P. canaliculata*. According to data from China’s Pesticide Information Network, 73 molluscicide products are officially registered, with common agents including metaldehyde and niclosamide sulfate, which exert molluscicidal effects through distinct mechanisms of action [7]. Metaldehyde exhibits relatively rapid efficacy. However, prolonged use has led to increased resistance in *P. canaliculata*, thereby reducing its control efficiency. Consequently, metaldehyde is often applied in combination with other compounds [8]. Although it is widely reported that metaldehyde exerts its molluscicidal activity via neurotoxic effects in *P. canaliculata*, this mechanism remains inadequately substantiated by diverse experimental approaches. Niclosamide sulfate, a derivative of vitamin B3 synthesized by condensation of nicotinic acid and aniline, has shown potential molluscicidal activity in laboratory studies under powder formulation [9]. Although this compound was studied as early as 30 years ago (e.g., as an active component in certain molluscicides), it remains unregistered in China’s Pesticide Information Network. Fentin acetate is registered as a fungicide in China and exhibits strong inhibitory effects on fungi. However, both niclosamide sulfate and fentin acetate have only been used as reference chemicals in screening experiments for molluscicides against *P. canaliculata* [10,11], and their efficacy and mechanisms of action against this snail species are not yet well understood.

*P. canaliculata* possesses a complete set of internal organs, several of which are critical for its environmental resilience. Its unique dual respiratory system, which includes a pulmonary sac, serves not only as an accessory breathing organ but also facilitates survival under desiccation or arid conditions. The kidney contributes to osmoregulation and the excretion of pesticide metabolites. The heart, characterized by a single ventricle and atrium, minimizes energy expenditure under low temperatures, aiding in environmental stress adaptation. Of particular relevance to this study, the liver serves as the primary organ for detoxification and immune defense, acting as the first line of response to physiological stress induced by chemical exposure [12].

Metabolomics enables the simultaneous qualitative and quantitative analysis of low-molecular-weight compounds, focusing on changes in endogenous metabolites following external stimuli or over time [13]. Because metabolites play central roles in regulating essential biological processes—including signal transduction, energy transfer, and homeostasis—their profiles provide an accurate reflection of cellular physiological states and environmental conditions. Non-targeted metabolomics, a comprehensive, high-throughput approach, provides an unbiased detection of the entire metabolite complement in biological samples. Compared with traditional targeted analyses, this approach has distinct advantages over traditional targeted analyses by systematically revealing the complex, thereby facilitating the discovery of new scientific discoveries [14].

Acetylcholinesterase (AChE) is a key enzyme involved in neural transmission, and its activity can serve as an indicator of neurotoxic effects. As a detoxification organ, changes in liver enzymes can better elucidate the toxic mechanisms of pesticides against *P. canaliculata* [15]. Superoxide dismutase (SOD) and catalase (CAT) are crucial members of the antioxidant enzyme system in biological organisms [16]. SOD activity indicates the capacity of an organism to scavenge oxygen-free radicals. Malondialdehyde (MDA), a primary end product of lipid peroxidation (LPO), serves as a marker for the extent of lipid peroxidation and associated cellular damage. Moreover, changes in MDA levels are closely correlated with alterations in SOD activity, with MDA serving as a reliable indicator of oxidative stress induced by free radicals [17].

This study aims to evaluate the toxicity of various chemical agents against *P. canaliculata* through indoor toxicity assays (immersion method) and to observe and analyze organ alterations in *P. canaliculata* following exposure. Preliminary findings indicate that the liver exhibits the most pronounced morphological changes under chemical stress. Given the liver’s central role as the primary detoxification organ, this study will investigate differences in liver metabolites of *P. canaliculata* after 12 h of exposure to a sublethal dose (LC_50_ concentration). Metabolomic approaches will be employed for qualitative and quantitative analysis of metabolites to elucidate potential metabolic pathways affected. Additionally, activities of key enzymes and biomarkers, including acetylcholinesterase (AChE), superoxide dismutase (SOD), catalase (CAT), and malondialdehyde (MDA), will be measured in the liver under chemical exposure to assess the induction of detoxification enzyme systems. A systematic, integrated analysis of phenotypic changes across different organs, alongside metabolite and enzyme activity alterations in the liver, will provide a comprehensive understanding of the organismal response. The significance of this study lies in elucidating the physiological and biochemical impacts of distinct chemical agents on *P. canaliculata* within the context of molluscicidal management, thereby providing a theoretical foundation for targeted management strategies against *P. canaliculata.*

## 2. Materials and Methods

### 2.1. Test Organisms

*P. canaliculata* individuals used in this study were collected using nets and cages from surrounding pools and ditches in Jingzhou District, Jingzhou City, Hubei Province, and subsequently reared in the laboratory. Following a one-week acclimatization period under uniform conditions, medium-sized individuals with an average weight of 4.0 ± 0.4 g were selected as experimental subjects. They were maintained in cylindrical tanks measuring 1.5 m in diameter, fed daily with cabbage, and the water was replaced with aerated water every two days. After the acclimatization period, the snails were used for subsequent experiments. There was no food intake during the experiment.

### 2.2. Toxicity Bioassay

The toxicity bioassay was conducted using the immersion method in accordance with the standard NY/T 1617-2008 “Guidelines for Laboratory Efficacy Testing of Molluscicides for Pesticide Registration” [18]. The tested agents included metaldehyde (ME), niclosamide sulfate (NS), and fentin acetate (FA). For each agent, pre-weighed amounts were thoroughly mixed with 1 L of aerated water in 1.5 L plastic containers (the lids of which were pre-punctured with small holes) and allowed to stand. The acclimatized *P. canaliculata*, grouped by weight (1.5–6.5 g per individual, medium size) with 20 individuals per replicate, was placed into the prepared containers. All containers were then maintained in a constant temperature chamber at 25 ± 2 °C and a relative humidity of 60 ± 5%. Based on preliminary experiments, five concentration gradients were established for each agent: 0.75, 1.5, 3, 6, and 12 mg/L. Each concentration was tested with four replicates. The behavioral status of the snails was observed every 12 h, and the number of dead individuals in each replicate was recorded, with dead snails being promptly removed. Mortality was determined using the following criterion: gently touching the exposed foot of the snail with forceps; active retraction indicated survival, while no response after multiple attempts was recorded as death.

Mortality and corrected mortality were calculated based on the observations, followed by probit regression analysis for toxicity evaluation. Mortality and corrected mortality were calculated using Formulas (1) and (2):

(1) $$\mathrm{Mortality}\;(\%)\;=\;\frac{\mathrm{Number}\;\mathrm{of}\;\mathrm{dead}\;\mathrm{Pomacea}\;\mathrm{canaliculata}}{\mathrm{Total}\;\mathrm{number}\;\mathrm{of}\;\mathrm{Pomacea}\;\mathrm{canaliculata}}\;\times \;100$$

(2) $$\mathrm{Corrected}\;\mathrm{mortality}\;(\%)=\frac{\mathrm{Mortality}\;\mathrm{in}\;\mathrm{treatment}\;\mathrm{group}-\mathrm{Mortality}\;\mathrm{in}\;\mathrm{control}\;\mathrm{group}}{1-\mathrm{Mortality}\;\mathrm{in}\;\mathrm{control}\;\mathrm{group}}\;\times \;100$$

### 2.3. Measurement of Organ Size Under Chemical Stress

The sizes of the liver, kidney, heart, pulmonary sac, and stomach of *P. canaliculata* were measured and calculated for comparative analysis. In subsequent experiments, medium-sized *P. canaliculata* (about 3.5 g) were exposed for 12 h to the 48 h LC_50_ concentrations of different agents using the immersion method. The swollen snails under toxic conditions were then dissected using anatomical tools. Dissections were performed in pre-prepared Petri dishes containing physiological saline kept on crushed ice. Different organs were placed on the culture dish and their length and width measured using a vernier caliper, and the surface area of each organ was calculated.

### 2.4. Determination of Non-Target Metabolites

Under 48 h LC_50_ exposure to three different agents, livers were dissected from *P. canaliculata* after 12 h of stress. A total of 24 liver samples (25–50 mg each) were collected, rinsed rapidly, and placed in tubes. The samples were divided into 4 groups with six replicates per group, flash-frozen in liquid nitrogen for 0.5 h, and then stored at −80 °C. Metabolomic analyses were conducted by Shanghai Majorbio Bio-pharm Technology Co, Ltd. (Shanghai, China) using the following platform instruments: QE-HFX (8 min), OE 240 (8 min), OE 480 (8 min), and TOF 6600 (12 min). Significantly different metabolites were considered based on the following criteria: a variable importance in projection (VIP) score ≥ 1 from the orthogonal partial least squares–discriminant analysis (OPLS-DA) model and a *p*-value < 0.05.

### 2.5. Detoxification Enzyme Activity Assay

*P. canaliculata* was exposed to the 48 h LC_50_ concentrations of three pesticides. After 12 h, the hepatopancreas was dissected, and its weight was accurately measured. A 1:9 (*w*/*v*) tissue homogenate was prepared by adding nine volumes of physiological saline (0.7% NaCl) per gram of tissue. The homogenate was centrifuged at 3000 rpm and 4 °C for 10 min, and the supernatant was collected for subsequent assays. The total protein (TP) concentration and the activities of acetylcholinesterase (AChE), superoxide dismutase (SOD), catalase (CAT), and malondialdehyde (MDA) in the liver were determined using commercial assay kits. The total protein assay kit (A045-2-2), malondialdehyde assay kit (A003-1-1), the acetylcholinesterase assay kit (A024-1-1), superoxide dismutase assay kit (A001-3-1), and catalase assay kit (A007-1-1) were all obtained from the Nanjing Jiancheng Bioengineering Institute (Nanjing, China).

### 2.6. Data Analysis

Statistical analyses were performed using SPSS 27.0 (SPSS Inc., Chicago, IL, USA). Median lethal concentrations (LC_50_) were obtained by using probabilistic analysis and dose–response data analysis, comparative analysis of organ surface area and enzyme activities was performed using one-way ANOVA, and Tukey’s post-hoc test was used for multiple comparisons of mean differences. Graphs were prepared using software GraphPad Prism 9.0 and Origin 2022.

## 3. Results

### 3.1. Toxicity of Three Chemicals on P. canaliculata

**Figure 1:**
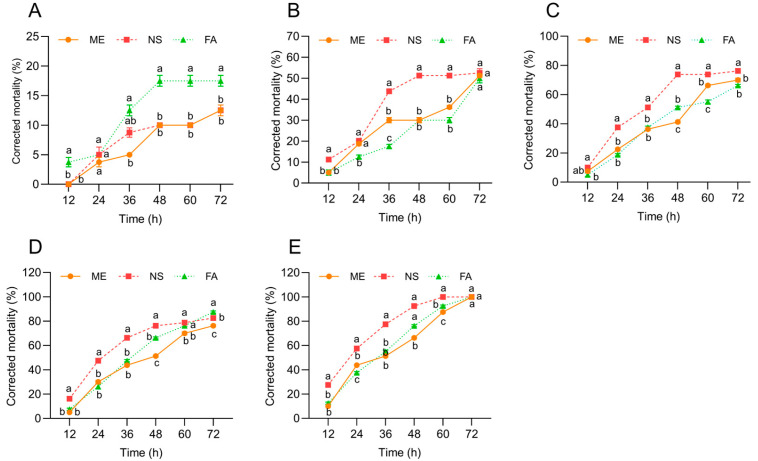
Toxicity effects of different indoor chemical agent treatment at concentrations of 0.75 mg/L^−1^ (**A**), 1.5 mg/L^−1^ (**B**), 3 mg/L^−1^ (**C**), 6 mg/L^−1^ (**D**) and 12 mg/L^−1^ (**E**) on *P. canaliculate*. Note: ME indicates metaldehyde; NS indicates niclosamide sulfate; FA indicates fentin acetate; data groups sharing the same letter indicate no significant difference between them (*p* > 0.05); whereas groups marked with different letters indicate a significant difference (*p* < 0.05); data are expressed as mean ± SE.

Significant differences were observed in the toxic efficacy of the three chemical agents against *P. canaliculata*. Corrected mortality increased progressively with rising pesticide concentrations. After 72 h of exposure at 12 mg·L^−1^, all three chemical agents resulted in 100% mortality (Figure 1E). At 0.75 mg·L^−1^, mortality remained below 20% for all agents; however, after 72 h, FA treatment caused significantly higher mortality than the other two agents (Figure 1A). Except for the 0.75 and 6 mg·L^−1^ (Figure 1A,D), NS treatment showed the highest toxicity against *P. canaliculate* (Figure 1). At the remaining concentrations, it generally induced significantly greater mortality compared with the other two agents. After 60 h of exposure, mortality rates for NS treatment reached 51.25%, 73.75%, 78.75%, and 100%, respectively. In contrast, under the same conditions, mortality rates for fentin acetate were 30%, 55%, 76.25%, and 92.5% and for metaldehyde 36.25%, 66.25%, 70%, and 84.5%, respectively (Figure 1).

The toxicity regression equations for *P. canaliculata* exposed to five concentrations of the three chemical agents for 24, 48, and 72 h are presented in Table 1. The results demonstrate that, among the three tested compounds, NS consistently exhibited the lowest LC_50_ values. After 24 h of exposure, the LC_50_ of all three chemical agents fell below 19 mg·L^−1^, with NS showing the lowest value (7.088 mg·L^−1^). By 48 h, the LC_50_ values of all agents declined to below 6 mg·L^−1^. Following 72 h of treatment, the LC_50_ values were 1.832 mg·L^−1^ for ME, 1.723 mg·L^−1^ for NS, and 1.903 mg·L^−1^ for FA. These findings indicate that NS possesses the highest toxicity toward *P. canaliculata*, whereas the toxicities of Fa and Me are relatively comparable.

### 3.2. Organ Changes in P. canaliculata by Three Chemicals Stressors

**Figure 2:**
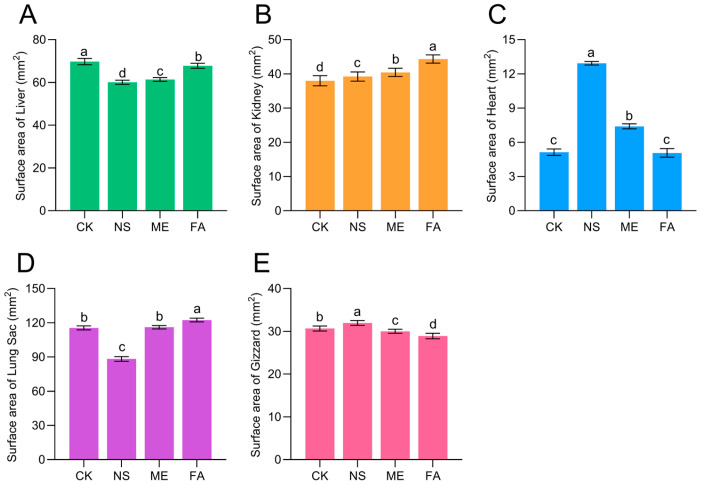
The surface area of the organs of *P. canaliculata* under different chemical stresses. Note: CK indicates control; ME indicates metaldehyde; NS indicates niclosamide sulfate; FA indicates fentin acetate; (**A**) surface area of liver; (**B**) surface area of kidney; (**C**) surface area of heart; (**D**) surface area of lung sac; (**E**) surface area of gizzard; data groups sharing the same letter indicate no significant difference between them (*p* > 0.05); whereas groups marked with different letters indicate a significant difference (*p* < 0.05); data are expressed as mean ± SE.

As shown in Figure 2, there was a significant change in the surface area of the organ of *P. canaliculate* after chemical agent treatment. The liver surface area of *P. canaliculata* was significantly reduced compared with the control (Figure 2A). The most pronounced reduction occurred under NS treatment, with a decrease of 9.71 mm^2^. In contrast, both the kidney and heart exhibited expansion. Kidney expansion was greatest following FA treatment, increasing by 6.36 mm^2^, whereas cardiac expansion was most marked under NS treatment, showing a highly significant difference from the control (Figure 2B,C). At the 48 h LC_50_ concentrations, the surface areas of the lung sac and stomach also varied. The lung sac surface area under NS treatment was significantly smaller than that of the control, while no significant difference was observed with ME treatment. FA treatment resulted in a lung sac surface area significantly larger than all other groups (Figure 2D). Regarding the gizzard, NS treatment induced significant expansion, while FA and ME treatments caused significant contraction. Significant differences were found among all four treatment groups (Figure 2E).

### 3.3. Sample Principal Component Analysis (PCA)

**Figure 3:**
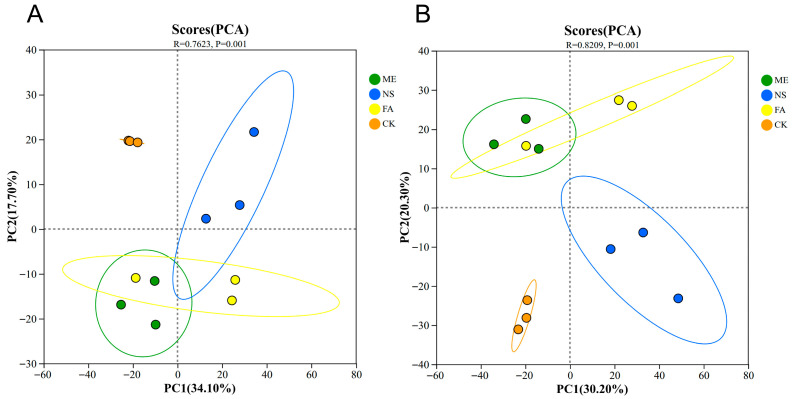
PCA plots of the control group and the ME, NS, and FA treatment groups in both anionic mode (**A**) and cationic mode (**B**), with confidence limits of 95%. Note: CK indicates control; ME indicates metaldehyde; NS indicates niclosamide sulfate; FA indicates fentin acetate; an R value closer to 1 indicates that the differences between groups are greater than the variation within groups, whereas a smaller R value suggests no distinct difference between groups relative to within-group variation. *p* = 0.001 indicates a statistically significant difference between the samples.

Principal component analysis (PCA) was conducted to evaluate the overall metabolic differences between groups and the within-group variability. In positive ion mode (Figure 3A), the first principal component (PC1) accounted for 30.20% of the total variance, and the second component (PC2) explained 20.30%. The model significance was confirmed (*p* = 0.001). In negative ion mode (Figure 3B), PC1 and PC2 contributed 34.10% and 17.70% of the total variance, respectively, also with a significant *p*-value of 0.001. These results demonstrate distinct inter-group differences in metabolite profiles.

### 3.4. Differential Metabolite Analysis

**Figure 4:**
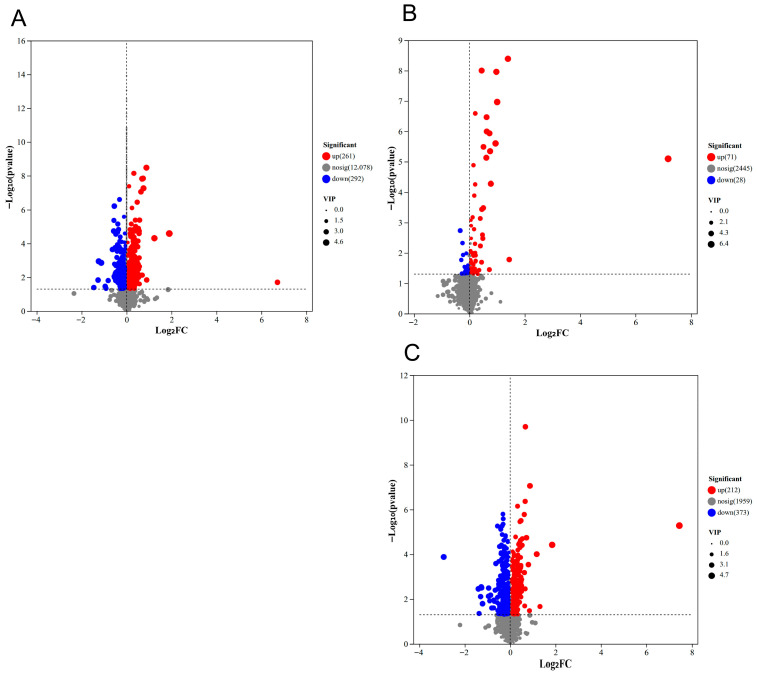
Volcano plot of differential metabolites between control group and ME (**A**), NS (**B**), and FA (**C**) treatments. Note: Metabolites above the horizontal dashed line are those with statistically significant differences, while those below are not significantly different; to the left of the vertical dashed line are significantly downregulated metabolites, and to the right are significantly upregulated ones.

A total of 553, 99, and 585 differential metabolites were identified in the ME, NS, and FA groups, respectively (Figure 4). In the ME group (Figure 4A), 261 metabolites were upregulated and 292 were downregulated. These differential metabolites were predominantly classified as lipids and lipid-like molecules, organic acids and derivatives, organoheterocyclic compounds, benzenoids, and organic oxygen compounds (Supplementary Figure S1). Among them, glutamine was significantly upregulated; acetic acid showed the most statistically significant change (lowest *p*-value); gilteritinib exhibited the greatest upregulation fold change; and sovitexin-2-O-arabinoside (FC = 0.3638) displayed the largest downregulation fold change. In the NS group (Figure 4B), compared with the control (CK) group, 71 metabolites were upregulated and 28 were downregulated. Gilteritinib was significantly upregulated; 4-aminophthalonitrile was the most significantly altered metabolite; gilteritinib again showed the largest regulation fold change; and pantetheine exhibited the greatest downregulation fold change. In the FA group (Figure 4C), 212 metabolites were upregulated and 373 were downregulated relative to CK. Gilteritinib was significantly upregulated; rebamipide was the most significantly altered metabolite; gilteritinib demonstrated the largest upregulation fold change; and p-mentha-1,3,5,8-tetraene showed the greatest downregulation fold change. All differential metabolites are listed in Supplementary Table S1.

### 3.5. KEGG Classification Analysis of Differential Metabolites

**Figure 5:**
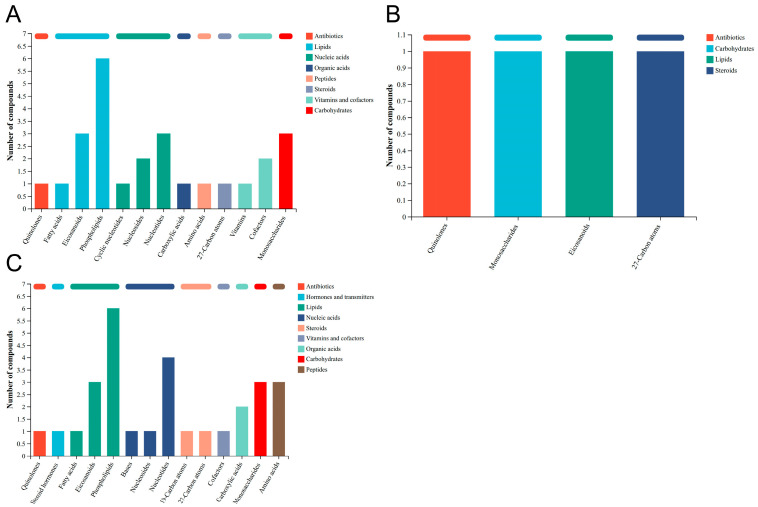
KEGG classification of differential metabolites in ME (**A**), NS (**B**), and FA (**C**) treatment.

KEGG analysis showed that differential metabolites were classified into organic acids, lipids, carbohydrates, nucleic acids, peptides, vitamins and cofactors, steroids, hormones and transmitters and antibiotics groups according to the KEGG path analysis (Supplementary Figure S2). In the ME group (Figure 5A), lipid metabolites—specifically eicosanoids and phospholipids—were significantly upregulated, with fold changes of 2.9 and 6.0, respectively, substantially impacting lipid metabolism pathways. While quinolones were also upregulated, the magnitude of increase was relatively lower. In the NS group (Figure 5B), a significant downregulation of eicosanoids affected lipid metabolism pathways, indicative of suppressed oxidative stress and inflammatory responses. In the FA group (Figure 5C), key lipid metabolites—including phospholipids, eicosanoids, steroid hormones, and fatty acids—were significantly altered. Additionally, nucleotides, bases, and nucleosides were associated with energy metabolism pathways.

### 3.6. KEGG Pathway Enrichment Analysis

**Figure 6:**
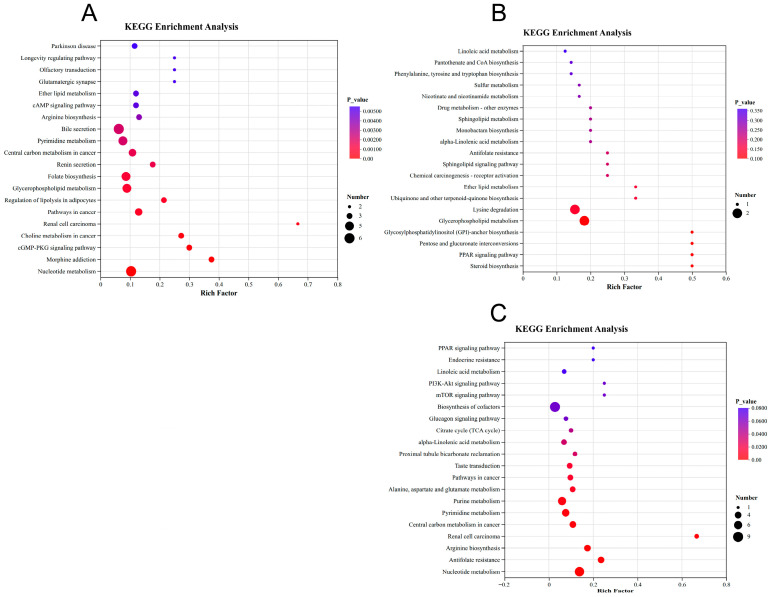
KEGG enrichment analysis of differential metabolites in ME (**A**), NS (**B**), and FA (**C**) treatment.

Bile secretion and nucleotide metabolism involved the largest number of differential metabolites, while glycerophospholipid metabolism and ether lipid metabolism exhibited significant activity in the ME group (Figure 6A). In the NS group (Figure 6B), lysine degradation and glycerophospholipid metabolism involved the highest number of metabolites, reflecting perturbations in lipid and amino acid metabolism. In the FA group (Figure 6C), the biosynthesis of cofactors included the most metabolites, with the peroxisome proliferator-activated receptor (PPAR) signaling pathway exhibiting the most pronounced enrichment, suggesting potential interference with lipid metabolism. Additionally, significant enrichment in purine metabolism, pyrimidine metabolism, and nucleotide metabolism indicates effects on cell division and tissue regeneration under FA treatment.

### 3.7. Effects of Chemical Exposure on Total Protein Content, Neurotoxicity and Oxidative Stress Biomarkers

**Figure 7:**
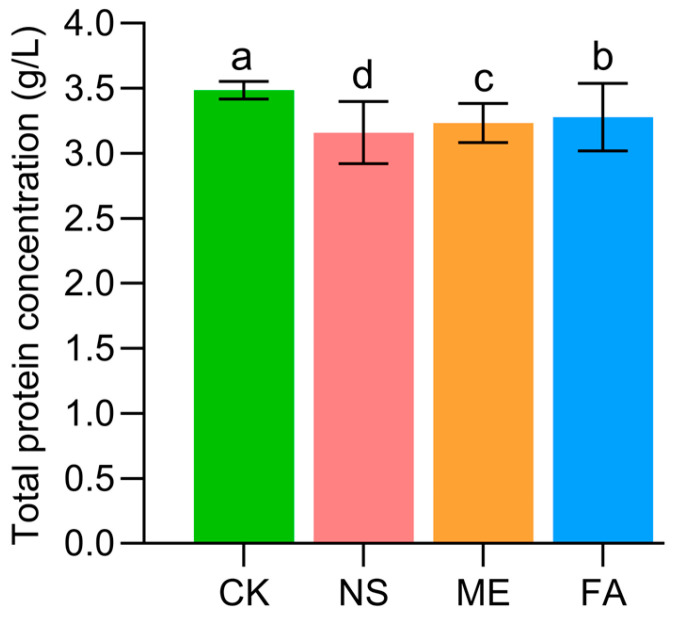
Effects of different agents on the total protein concentration in the liver of *P. canaliculata.* Note: CK indicates control; ME indicates metaldehyde; NS indicates niclosamide sulfate; FA indicates fentin acetate; data groups sharing the same letter indicate no significant difference between them (*p* > 0.05); whereas groups marked with different letters indicate a significant difference (*p* < 0.05); data are expressed as mean ± SE.

**Figure 8:**
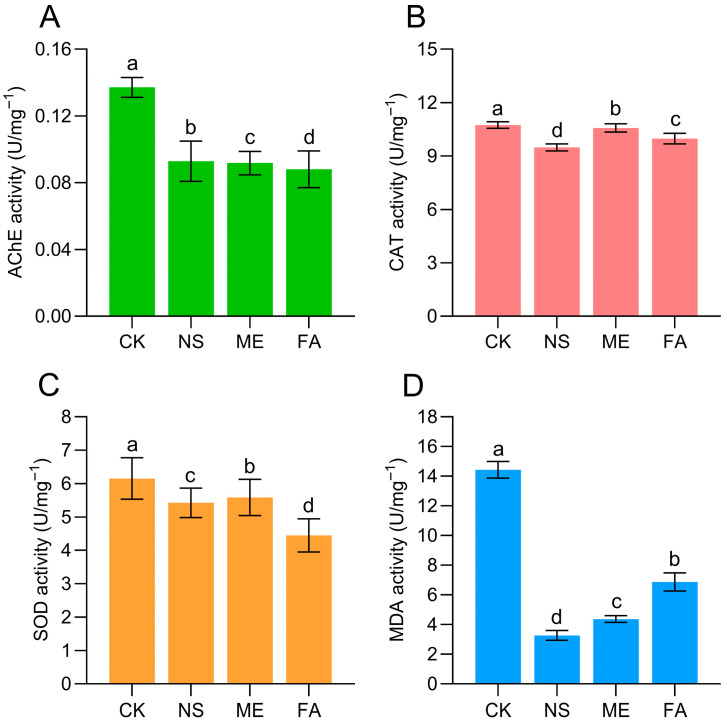
Effects of different agents on the activity of AChE (**A**), CAT (**B**), SOD (**C**) and MDA (**D**) in the liver of *P. canaliculata.* Note: CK indicates control; ME indicates metaldehyde; NS indicates niclosamide sulfate; FA indicates fentin acetate; data groups sharing the same letter indicate no significant difference between them (*p* > 0.05); whereas groups marked with different letters indicate a significant difference (*p* < 0.05); data are expressed as mean ± SE.

Total protein (TP) concentration in the liver of *P. canaliculata* was significantly reduced under exposure to three chemical agents, especially in NS treatment, which resulted in the lowest concentration at 3.16 g/L (Figure 7). Activities of acetylcholinesterase (AChE), catalase (CAT), and superoxide dismutase (SOD) were also significantly inhibited across the treatment groups, accompanied by reduced malondialdehyde (MDA) levels (Figure 8). Specifically, AChE and MDA activities were markedly lower compared with the control (Figure 8A,D). FA group resulted in the lowest AChE activity, and the lowest MAD activity was exhibited in NS group. The lowest SOD activity was shown in the ME group, with activities in the NS and FA groups being significantly higher. For CAT activity, no significant difference was found between the control group and the other groups, except for a notable difference compared to the NS group for ME and FA control. Overall, these results indicate that all three chemical agents suppressed hepatic AChE, CAT, and SOD activities and reduced MDA levels in *P. canaliculata*.

## 4. Discussion

The median lethal concentrations (LC_50_) of metaldehyde (ME), niclosamide sulfate (NS), and fentin acetate (FA) against *P. canaliculata* were determined at multiple time intervals, providing comparative toxicity profiles for these compounds (Table 1). Following the observation of body swelling in snails after 12 h of exposure, morphological and histological alterations in internal organs under chemical stress were further examined (Figure 2). Focusing on the hepatopancreas, a central organ for detoxification and immune regulation, we measured the activities of acetylcholinesterase (AChE), superoxide dismutase (SOD), catalase (CAT), and malondialdehyde (MDA) (Figure 8) and performed non-targeted metabolomic profiling in snails exposed to LC_50_ concentrations of the three agents for 12 h. These results advance the understanding of the toxic mechanisms of these chemicals from a detoxification and metabolic perspective and provide a theoretical basis for the chemical control of *P. canaliculata* in field applications.

Among the chemical agents evaluated in this study, only metaldehyde is registered as a molluscicide according to the China Pesticide Information Network [19]. The other two compounds are not registered for molluscicidal application. Specifically, niclosamide sulfate remains unregistered on the China Pesticide Information Network, while fentin acetate is listed solely as a fungicide. Notably, both niclosamide sulfate and fentin acetate exhibited higher toxicity to *P. canaliculata* within 48 h compared to metaldehyde (Figure 1). As an early-generation molluscicide, prolonged application of metaldehyde may have led to the development of resistance in *P. canaliculata*, potentially inducing the expression of resistance-related genes over successive generations [20]. Although the other two chemical agents demonstrated considerable molluscicidal effects against *P. canaliculata* in the experiment, their potential environmental risks remain uncertain, as there is currently no experimental evidence to assess their ecological safety.

Analysis of the poisoning patterns and physiological states of *P. canaliculata* in this study revealed that the majority of individuals exhibited body swelling following chemical stress. This phenomenon resembles the liver swelling previously reported in *P. canaliculata* under benzoquinone molluscicide stress [21]. As the primary detoxification organ in this species, the liver may undergo significant morphological and functional alterations under chemical stress. Consistently, our results demonstrated a marked reduction in liver surface area across all chemical treatments (Figure 2A), in agreement with observations reported by Gao et al. [22], likely reflecting the high sensitivity of hepatic tissue to these chemicals. In addition, the kidney, as an organ responsible for excreting metabolic waste, showed significant enlargement after chemical treatment (Figure 2B). The heart, which plays an auxiliary role in respiration in *P. canaliculata*, exhibited enlargement under treatment with niclosamide sulfate and metaldehyde (Figure 2C), indirectly suggesting that these two chemicals may interfere with respiratory function. As a pulmonate snail, *P. canaliculata* regulates buoyancy through active inhalation or expulsion of air via the lung sac [23]. Exposure to fentin acetate, the lung sac of *P. canaliculata* exhibited enlargement (Figure 2D), likely as a defensive response to chemical stress, aiming to reduce the inhalation of additional chemicals during respiration. In contrast, niclosamide sulfate treatment led to a reduction in lung sac size (Figure 2D), indicating that this chemical may exert its control effect by inhibiting the respiration of *P. canaliculata*.

Under exposure to different chemical agents, the total protein (TP) concentration in the liver of *P. canaliculata* decreased to varying degrees (Figure 7), consistent with previous reports [24]. This observation suggests that chemical agent stress may interfere with the normal metabolic processes of *P. canaliculata*, which is consistent with the results obtained from metabolomics analysis. Meanwhile, the activity of AChE in the liver was significantly reduced under chemical agent stress (Figure 8), indicating that the chemical agent may exert its control effects by inhibiting the nervous system of *P. canaliculata* [25]. Additionally, the activities of CAT, SOD, and MDA decreased to different extents (Figure 8), reflecting a stress response in *P. canaliculata* under drug exposure. This may be attributed to the activation of the antioxidant system to mitigate oxidative damage and initiate self-repair processes [26,27,28,29].

AChE levels decreased across three groups (Figure 8), with the most pronounced decline observed in the metaldehyde group. The reduction in AChE indicates potential neurotoxicity. In the metaldehyde group, changes in the metabolite phosphocholine, a key precursor for acetylcholine and phospholipid synthesis, corroborated AChE inhibition and membrane damage [30]. Additionally, the activation of kynurenine and adenosine pathways, closely associated with neuroinflammation and oxidative stress, likely reflects compensatory responses to energy deficits and excitotoxicity (Figure 4A) [31]. Decline in CAT and SOD activities pointed to the collapse of the antioxidant system [32]. This interpretation is further supported by metabolite changes in the metaldehyde group, including glutamine, cystine, riboflavin, flavin mononucleotide, and lipoic acid, as well as in the niclosamide sulfate group, with metabolites such as pantetheine and s-acetyldihydrolipoamide (Figure 4). These metabolites are established markers of antioxidant system depletion and oxidative damage, aligning fully with the changes in oxidative stress indicators (CAT, SOD, and MDA) [33]. The observed decrease, rather than the anticipated increase, in MDA levels may be attributable to the relatively low LC_50_ concentrations used and the short exposure duration [34]. Overall, this study provides insights into the physiological responses on various chemical agents, which demonstrated promising molluscicidal effects against *P. canaliculata*. However, the non-molluscicidal agents used in this study have not yet undergone systematic environmental safety assessment and testing. Subsequent research involving ecotoxicological evaluation could provide a scientific basis for optimizing application strategies of these agents, thereby effectively enhancing their control efficacy against *P. canaliculata* while minimizing potential ecological risks.

## 5. Conclusions

This study indicates that the various chemical agents examined exert differential regulatory effects on the detoxification metabolism and lethal mechanisms in the invasive snail species *P. canaliculata*. These substances influence *P. canaliculata’s* survival through multiple pathways. Organ-specific analyses revealed that niclosamide sulfate affected the size of the pulmonary sac, thereby impairing respiratory function, whereas inhibition of acetylcholinesterase activity in the hepatopancreas inferred that metaldehyde may interfere with neural transmission in the *P. canaliculata*. Furthermore, metabolomic analysis revealed a substantial number of differentially abundant metabolites in the hepatopancreas following chemical exposure, many of which are associated with the depletion of the antioxidant system and disruption of lipid metabolism. These findings provide a theoretical foundation for the development of novel and highly effective molluscicides and also possess practical application value. Moreover, the results indicate that unregistered chemicals such as niclosamide sulfate and triphenyltin acetate exhibit significant toxicity against *P. canaliculata*, underscoring their potential as promising molluscicidal candidates.

## Figures and Tables

**Table 1:** Toxicity of three chemical agents against *P. canaliculata*, with confidence limits of 95%.

Chemical Agent	Treatment Time (h)	Virulence Regression Equation	LC_50_ (mg/L^−1^)	Slope ± SE	95% Confidence Interval	Correlation Coefficient (r)
ME	24	y = −1.371 + 1.142x	15.871	1.142 ± 0.365	7.772~193.495	0.973
	48	y = −0.900 + 1.270x	5.018	1.270 ± 0.330	3.174~10.987	0.952
	72	y = −0.601 + 2.284x	1.832	2.284 ± 0.420	1.273~2.465	0.934
NS	24	y = −1.188 + 1.397x	7.088	1.397 ± 0.350	4.487~16.879	0.953
	48	y = −0.711 + 1.667x	2.669	1.667 ± 0.345	1.738~3.935	0.916
	72	y = −0.586 + 2.481x	1.723	2.481 ± 0.449	1.219~2.283	0.954
FA	24	y = −1.293 + 1.014x	18.822	1.014 ± 0.358	8.214~807.208	0.939
	48	y = −0.775 + 1.431x	3.480	1.431 ± 0.331	2.217~5.760	0.986
	72	y = −0.297 + 1.852x	1.903	2.357 ± 0.427	1.349~2.543	0.953

Note: x represents the logarithm of the concentration of the treated solution, and y represents the probability value of the treatment mortality rate.

## Data Availability

All original data and materials presented in this study are included in the article and Supplementary Materials. Further inquiries can be directed to the corresponding authors.

## Supplementary Materials

The following supporting information can be downloaded at: https://www.mdpi.com/article/10.3390/biology15070529/s1, Figure S1: Correlation heat map of ME (A), NS (B), FA (C) and CK differential metabolites; Figure S2: Compound classification of all metabolites; Table S1: All differential metabolites.
