# Supplementary figures and images for: Toxicity Effects and Mechanism of Chemical Stress on Pomacea canaliculata
Source: Biology (Basel). 2026 Mar 26;15(7):529. doi: 10.3390/biology15070529 (PMC13072044; doi:10.3390/biology15070529)

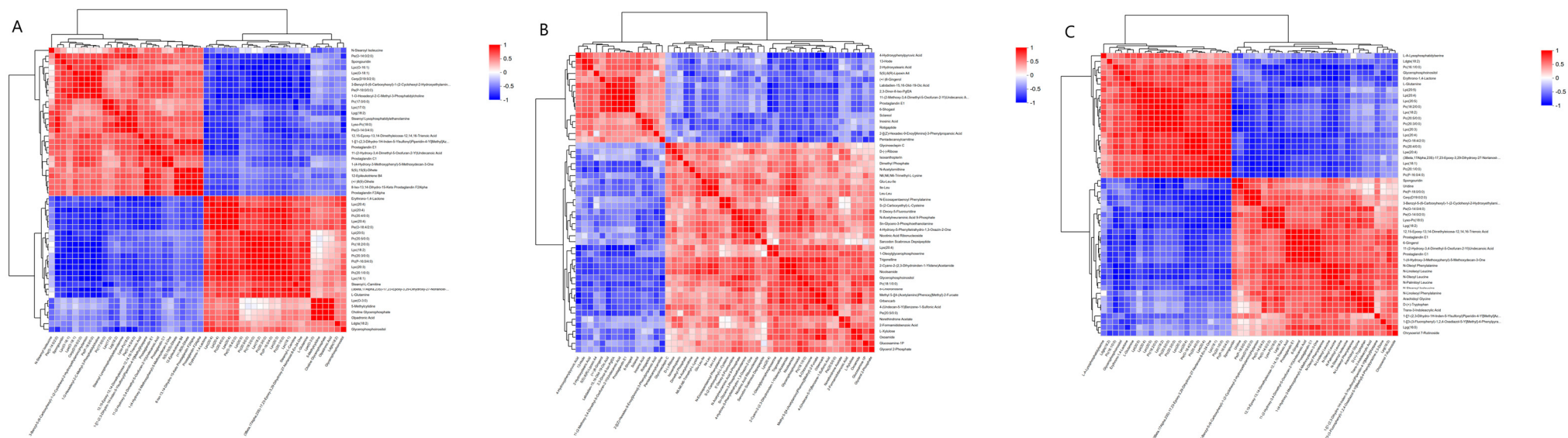

**Figure S1.** Correlation heat map of ME (A), NS (B), FA (C) and CK differential metabolites

Supplement: Supplementary file 1 [file biology-15-00529-s001.zip › Figure S1 Correlation heat map of ME (A), NS (B), FA (C) and CK differential metabolites.pdf]

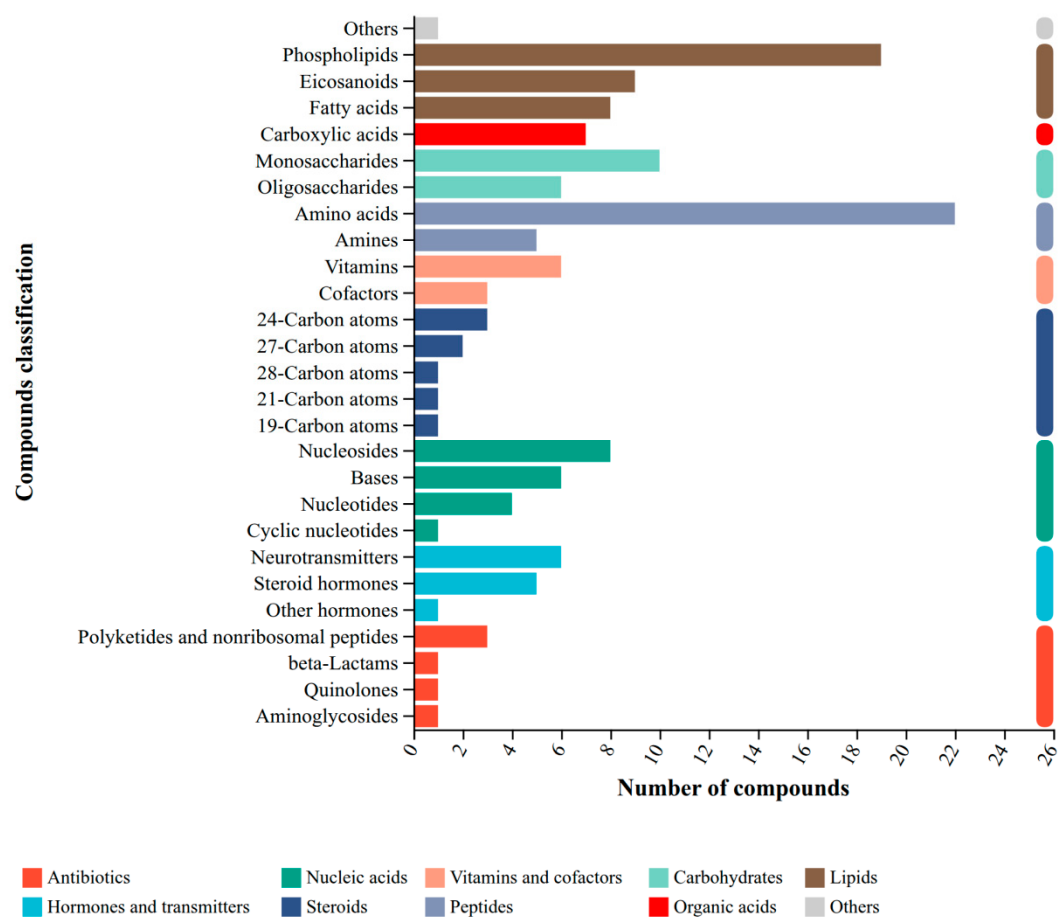

**Figure S2.** Compounds classification of all metabolites.

Supplement: Supplementary file 1 [file biology-15-00529-s001.zip › Figure S2 Compounds classification of all metabolites.pdf]
